# Elamipretide effects on the skeletal muscle phosphoproteome in aged female mice

**DOI:** 10.1007/s11357-022-00679-0

**Published:** 2022-11-02

**Authors:** Matthew D. Campbell, Miguel Martín-Pérez, Jarrett D. Egertson, Matthew J. Gaffrey, Lu Wang, Theo Bammler, Peter S. Rabinovitch, Michael MacCoss, Wei-Jun Qian, Judit Villen, David Marcinek

**Affiliations:** 1 Department of Radiology, University of Washington, South Lake Union Campus, 850 Republican St., Brotman D142, Box 358050, Seattle, WA 98109 USA; 2 Department of Genome Sciences, University of Washington, Seattle, WA USA; 3 Biological Sciences Division, Pacific Northwest National Laboratory, Richland, WA USA; 4 Department of Environmental and Occupational Health Sciences, University of Washington, Seattle, WA USA; 5 Department of Laboratory Medicine and Pathology, University of Washington, Seattle, WA USA

**Keywords:** Aging, Mitochondria, Sarcopenia, Phosphorylation, Proteomics, S-Glutathionylation

## Abstract

The age-related decline in skeletal muscle mass and function is known as sarcopenia. Sarcopenia progresses based on complex processes involving protein dynamics, cell signaling, oxidative stress, and repair. We have previously found that 8-week treatment with elamipretide improves skeletal muscle function, reverses redox stress, and restores protein S-glutathionylation changes in aged female mice. This study tested whether 8-week treatment with elamipretide also affects global phosphorylation in skeletal muscle consistent with functional improvements and S-glutathionylation. Using female 6–7-month-old mice and 28–29-month-old mice, we found that phosphorylation changes did not relate to S-glutathionylation modifications, but that treatment with elamipretide did partially reverse age-related changes in protein phosphorylation in mouse skeletal muscle.

**Supplementary Information:**

The online version contains supplementary material available at 10.1007/s11357-022-00679-0.

## Introduction

Aging is a complex process involving genetic, molecular, protein, and cellular interactions [1]. Skeletal muscle aging is a particularly important and dynamic process due to the necessity of a well-functioning musculoskeletal system for maintaining mobility and metabolic homeostasis [2]. Sarcopenia is the age-related loss of skeletal muscle strength and mass [3]. Development and progression of sarcopenia has been linked to changes in hormones, energy production, inactivity, neuromuscular junction dysfunction, mitochondrial decline, and redox status [4–8]. In order to maintain quality of life in a rapidly aging population [9], it is necessary to identify treatments to combat the development and progression of sarcopenia.

Elamipretide (ELAM, formerly Bendavia and SS-31) is a short tetrapeptide that interacts with cardiolipin and cardiolipin-interacting proteins on the inner mitochondrial membrane [10, 11]. In aged female mice, ELAM given both acutely and long term can improve in vivo mitochondria function, increase fatigue resistance, and restore redox status in skeletal muscle [12, 13]. Other studies in mice have shown that ELAM improves cardiac diastolic function in males and females [14, 15], visual function in females and males [16], cognitive function in males [17], and kidney glomerular architecture in females and males [18]. These functional improvements are supported by direct interaction of ELAM with mitochondrial proteins responsible for mitochondrial ATP production, metabolism, and signaling [11]. Despite the demonstrated reversal of mitochondrial dysfunction and the associated functional improvements, the cellular mechanisms linking mitochondrial function to reversal of aging pathology with ELAM remain poorly defined.

One of the most profound effects of ELAM in skeletal muscle is its ability to restore cellular redox status [13]. As muscle ages, oxidative damage increases and redox homeostasis is disrupted due to a two-pronged increase in dysregulation of antioxidant systems, and production of oxidants through metabolic processes [19]. These defects in oxidant production and clearance cause increased oxidative post-translational modification of cysteine residues with age [13, 20]. Previous work demonstrated that 8-week treatment with ELAM restores global cysteine S-glutathionylation in aged muscle to a profile that more closely resembles that of young muscle [13]. This comprehensive and remarkable change in global cysteine oxidation led to the hypothesis that restoring mitochondrial function in aged skeletal muscle with ELAM treatment would reverse age-related changes in other transient and reversible signaling systems such as phosphorylation.

This study was designed to investigate whether ELAM could alter protein expression or post-translational modifications in skeletal muscle. This study was performed in females for two reasons: (1) the desire to link changes in the proteome to our previous data showing improved mitochondrial bioenergetics and muscle function in females [13], and (2) we wanted to directly compare the effects on the phosphoproteome to our previous results on protein S-glutathionylation in the same muscle samples. To accomplish this, we treated aged female mice with ELAM for 8 weeks and measured changes in protein abundance and the phosphoproteome compared to untreated age matched controls and untreated young animals.

## Methods

### Animals and design

All experiments in this study were reviewed and approved by the University of Washington Institutional Animal Care and Use Committee. Mice used were female C57BL/6 received from the National Institute of Aging aged rodent colony. Mice were maintained at 21 °C on a 14/10 light/dark cycle and given standard mouse chow and water ad libitum without deviation throughout 8-week treatment and prior to euthanasia, dissection, and experimental procedures. Six young animals were sacrificed at 6–7 months of age. Seven aged saline untreated and seven aged elamipretide-treated mice were sacrificed at 28–29 months of age. Following 8-week ELAM treatment, animals were euthanized using cervical dislocation and gastrocnemius muscle was dissected from animals and split into three portions for (1) global abundance proteomics, (2) phosphoproteomics, and (3) S-glutathionylation proteomics.

### Surgery and elamipretide

ELAM was prepared in isotonic sterile saline and loaded into osmotic pumps (Alzet #1004) to deliver 3 mg·kg^−1^·day^−1^. Aged animals were induced for anesthesia using 4% isoflurane in 1 L/min O_2_ and maintained during surgery at 1.5–2% isoflurane on a circulating water pad at 37 °C. A 1-cm incision was made along the midback, and pumps were implanted subcutaneously. The incision was stapled shut using two 7-mm wound clips and a drop of Vetbond tissue glue (3 M). Animals were injected intraperitoneally with supplemental meloxicam for pain management at 5 mg/kg prior to cessation of anesthesia. Animals were allowed to recover in a heated cage and monitored daily following surgery for 7 days until surgical incisions had healed. At 4 weeks post-surgery, the animals underwent surgery again as described to remove the old pump and replace with a newly primed and ELAM loaded pump.

### Sample preparation for phosphoproteomics

Approximately 75 mg of snap-frozen gastrocnemius muscle was ground with a mortar and pestle and resuspended in lysis buffer (8 M urea, 75 mM NaCl, 50 mM Tris, pH 8.2, with Roche Complete EDTA-free protease inhibitors and phosphatase inhibitors 50 mM beta-glycerophosphate, 50 mM sodium fluoride, 10 mM sodium pyrophosphate, and 1 mM sodium orthovanadate). Zirconia beads (0.5 mm) were added to lysates and bead beat using a bullet blender (Next Advance, Troy, NY) on high for 1 min followed by sonication on ice for 5 min. Lysates were centrifuged at 4 °C. Samples were reduced using 5 mM dithiotreitol at 55 °C for 30 min followed by alkylation using 15 mM iodacetamide at room temperature in the dark for 30 min. Quenching was performed using 5 mM dithiotreitol at room temperature for 15 min. Samples were then diluted 1:5 with 50 mM Tris pH 8.2 and proteolytic digestion proceeded using trypsin at 1:200 (enzyme/protein) at 37 °C overnight. Quenching of digestion proceeded with trifluoroacetic acid addition to pH 2.0. Peptide samples were centrifuged and desalted using a 50-mg tC18 SepPak cartridge (Waters Corp, Milford, MA) as previously described [21]. Then, 20- and 500-µg aliquots of eluted peptides were dried by vacuum centrifugation and stored at − 80 °C for proteomic and phosphoproteomic analysis, respectively.

### Phosphoproteomics

Dried peptides were resuspended in 80% acetonitrile and 1% trifluoroacetic acid, and enriched for phosphopeptides via immobilized metal affinity chromatography as previously described. LC–MS/MS was performed as previously described [21, 22].

### Proteomics

Samples for proteomics were prepared as previously described [13]. Briefly, approximately 10–20 mg of snap-frozen tissue was lysed and digested in 50 mM ammonium bicarbonate and homogenized using a Bullet Blender (Next Advance, Troy NY). Following, lysis samples were acidified and cleaned for liquid chromatography using MCX columns (Waters) and mass spectrometry was performed as previously described [13].

### S-Glutathionylation proteomics

Samples for profiling of S-glutathionylation were prepared as previously described [13]. In brief, approximately 25–50 mg of frozen mouse gastrocnemius was homogenized in the presence of *n*-ethyl-maleimide (NEM) to block free thiols. NEM was omitted from homogenization buffer for generation of samples intended for total thiol analysis. Homogenization was performed using a handheld homogenizer until tissue was completely homogenized. Samples were acetone precipitated overnight and washed three times to remove excess NEM. The resulting protein pellet was solubilized and subjected to three rounds of buffer exchange before proteins were selectively reduced using GRX1 enzyme cocktail. Samples intended for total thiol analysis were reduced using dithiotreitol (DTT). Following reduction, samples were enriched using Thiopropyl Sepharose 6B resin for SSG-modified proteins and total thiols using 564 and 250 µg of protein, respectively. After on-resin tryptic digestion and isobaric labeling using 10-plex tandem mass tag (TMT) reagents (Thermo Fisher), peptides were eluted and LC–MS/MS was performed using a Q-Exactive Plus (Thermo Fisher).

### Data analysis

Proteomics datasets were analyzed using MSStats Skyline pipeline as previously described [23]. Heatmaps were generated using computed *z*-scores of log_2_-transformed total signal. Ingenuity Pathway Analysis (IPA) core analysis (Expression Analysis) was performed on proteins with a false discovery rate (FDR) of 0.1 as cutoff. Phosphoproteomics datasets were analyzed using log_2_ median-normalized intensity data. IPA core analysis (Phosphorylation Analysis) was performed on sites with an FDR < 0.1. S-Glutathionylation proteomics datasets were analyzed using log_2_ median-normalized intensity data. IPA core analysis (Expression Analysis) was performed with an FDR < 0.1.

## Results

### Global proteomics and S-glutathionylation proteomics

**Fig. 1:**
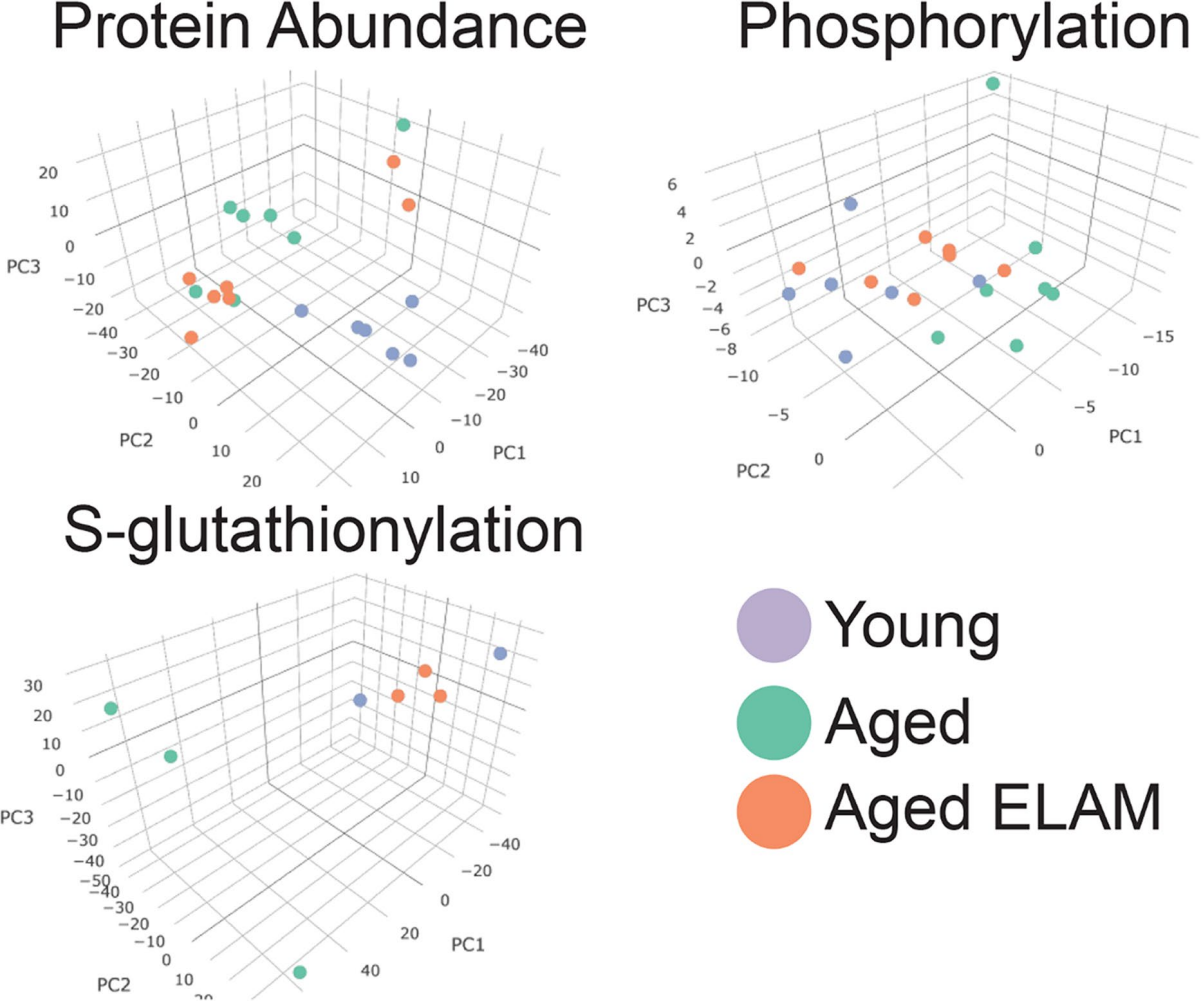
Principal component analysis of protein abundance, phosphorylation, and S-glutathionylation proteomics

We compared 5-month-old female mice to 28-month-old female mice treated for 8 weeks with saline or ELAM. We previously published summary data showing that 8-week treatment with ELAM reverses age-related S-glutathionylation of the skeletal muscle thiol proteome [13]. Here, we provide additional analyses of these data on S-glutathionylation in the context of changes in the phosphoproteome from the same muscle. Global protein abundance analysis identified 1679 unique proteins. Using a false discovery rate (FDR) of < 0.1, we identified 43 proteins that were significantly different in skeletal muscle between aged and young mice. We did not identify any significant changes in the proteome of skeletal muscle of aged mice by treatment with ELAM. Three-dimensional principal component analysis (PCA) of log_2_-transformed, normalized intensities of proteins in young, aged, and aged ELAM-treated animals showed that aged ELAM treated animals cluster more closely to aged animals than young animals (Fig. 1).

S-Glutathionylation analysis identified 5526 unique cysteine sites on 1948 proteins. Using FDR of < 0.1 revealed 1490 cysteine residues that were altered between aged and young mice. Treatment with ELAM in aged mice altered 2691 residues compared to aged mice. Three-dimensional PCA showed strong clustering of the aged ELAM-treated mice with young animals that were well separated from aged untreated animals (Fig. 1).

### Phosphoproteomics

**Fig. 2:**
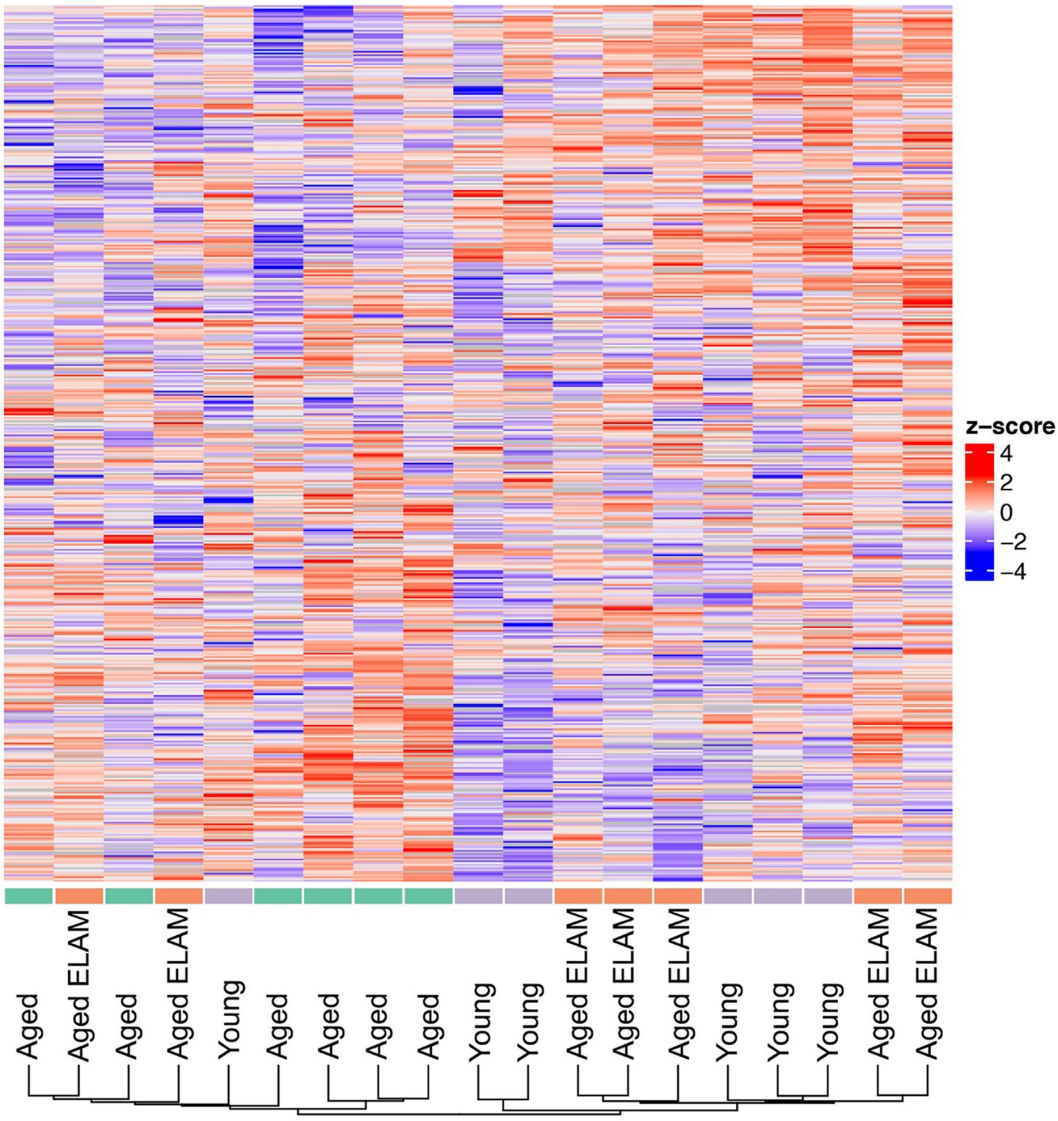
Phosphorylation site intensities. All measured phosphorylation sites showing < 30% missing values in samples of young, aged, and aged ELAM-treated animals

**Fig. 3:**
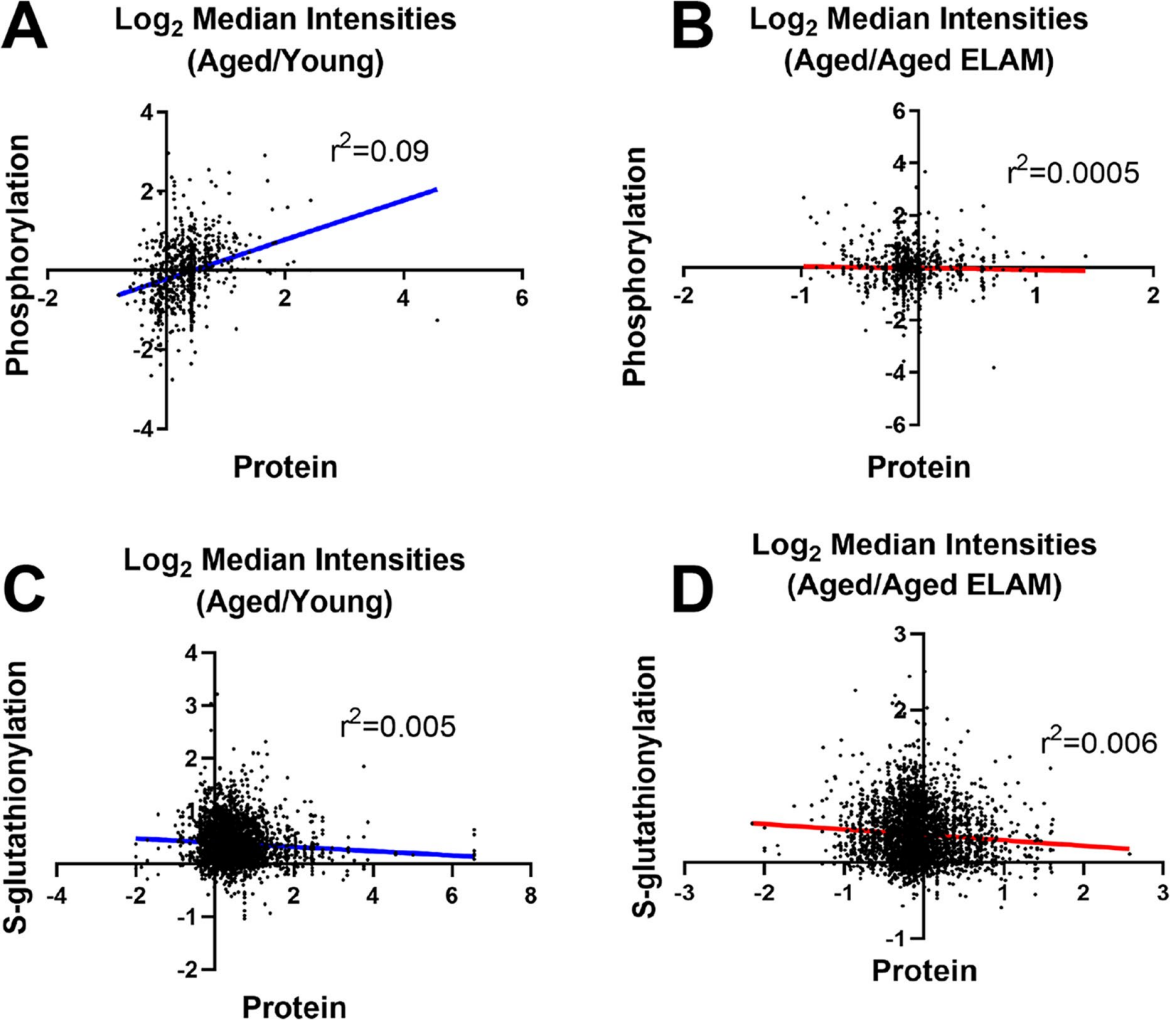
Comparison of phosphorylation and S-glutathionylation changes with changes in protein abundance. **A** Aged/young comparison of phosphorylation to protein abundance. **B** Aged/aged ELAM comparison of phosphorylation to protein abundance. **C** Aged/young comparison of S-glutathionylation to protein abundance. **D** Aged/aged ELAM comparison of S-glutathionylation to protein abundance. For proteins with multiple phosphorylation sites, each phosphorylation site or cysteine residue is an individual data point

**Fig. 4:**
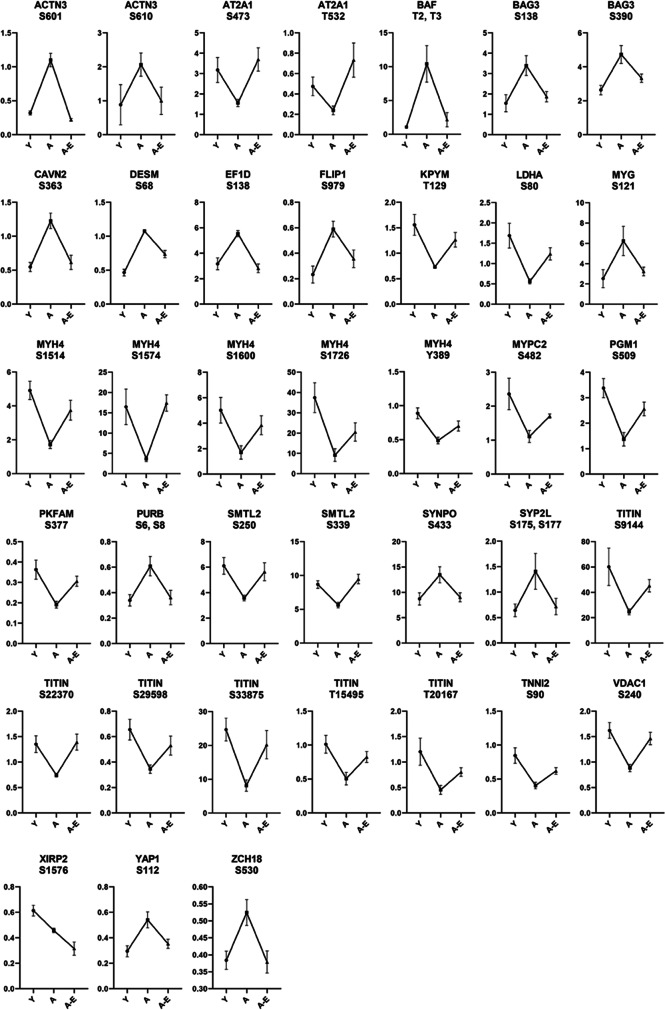
Median intensities of 38 phosphorylation sites. All sites are significantly altered in both aged/young and aged/aged ELAM-treated comparisons. *Y*-Axis is the median intensity of the selected residue. *X*-Axis labels: *Y* young, *A* aged, *A-E* aged ELAM treated. One-way ANOVA *p* < 0.05

Phosphorylated peptide analysis identified 1541 unique phosphorylation sites on 515 unique proteins. Using FDR of < 0.1 revealed six phosphorylation sites that were altered between aged and young mice and a single phosphorylation site between aged and aged ELAM-treated mice. Accordingly, the three-dimensional PCA of log_2_-transformed, normalized phosphorylation sites did not show any clear group separation (Fig. 1). Heatmap cluster analysis of all phosphorylation sites with fewer than 30% missing values shows modest clustering of muscles from aged ELAM-treated mice with muscles of young mice (Fig. 2), indicating that ELAM tends to reverse age-related changes in the phosphoproteome. However, these effects are more subtle than previously reported for S-glutathionylation [13]. Comparison of the phosphoproteomic datasets indicates that the phosphorylation signal does not correlate with changes in protein abundance (Fig. 3). Changes in S-glutathionylation were also not correlated with changes in protein abundance (Fig. 3B). Thus, the altered post-translational modifications identified with age and ELAM treatment were not driven by changes in protein abundance. ANOVA with a Tukey correction for multiple comparisons of individual phosphorylation sites identified 38 residues that were significantly altered with both age and ELAM treatment (*p* < 0.05). In all but one site, on the Xin Actin-Binding Repeat-Containing 2 (XIRP2) protein, ELAM treatment reversed the age-related direction of changes in phosphorylation with age (Fig. 4). Gene ontology analysis of significantly altered phosphorylation sites (ANOVA, *p* < 0.05) was performed to investigate both cellular component (Table 1) and biological process (Table 2).

**Table 1:** Gene ontology breakdown of significant phosphorylation changes by cellular component

	Aged/young (145)	Aged/aged ELAM (90)	Both (38**)**
1	Intracellular (132)	Cell (84)	Intracellular (36)
2	Cell (132)	Cell part (84)	Cell (26)
3	Intracellular part (132)	Intracellular (83)	Organelle (36)
4	Cell part (132)	Intracellular part (83)	Intracellular part (36)
5	Organelle (127)	Organelle (81)	Cell part (36)
6	Cytoplasm (120)	Cytoplasm (78)	Cell part (36)
7	Intracellular organelle (119)	Intracellular organelle (78)	Cytoplasm (33)
8	Cytoplasmic part (109)	Organelle part (70)	Intracellular organelle (33)
9	Organelle part (107)	Cytoplasmic part (68)	Organelle part (31)
10	Non-membrane-bounded organelle (99)	Intracellular organelle part (62)	Cytoplasmic part (31)
Nucleus	64	40	18
Mitochondrion	13	9	5
Endoplasmic reticulum	9	7	3
Golgi	1	1	0
Vesicle	53	29	15
Cytoskeleton	83	47	20
Plasma membrane	36	29	10
Cytosol	33	24	12
Myofibril	78	42	23
Extracellular region	52	28	15

Ten most frequent cellular components and selection of major skeletal muscle organelles are shown. Number of residues significantly altered are shown in parentheses and for selected processes. One-way ANOVA *p* < 0.05

**Table 2:** Gene ontology breakdown of significant phosphorylation changes by biological process

	Aged/young (145)	Aged/aged ELAM (90)	Both (38)
1	Cellular process (124)	Cellular process (74)	Cellular process (34)
2	Single-organism process (113)	Single-organism process (73)	Single-organism process (32)
3	Single-organism cellular process (110)	Single-organism cellular process (65)	Single-organism cellular process (30)
4	Biological regulation (102)	Biological regulation (62)	Biological regulation (28)
5	Regulation of biological process (100)	Multicellular organismal process (60)	Regulation of biological process (27)
6	Regulation of cellular process (98)	Regulation of biological process (60)	Regulation of cellular process (27)
7	Multicellular organismal process (92)	Single-multicellular organism process (56)	Multicellular organism process (26)
8	Response to stimulus (88)	Regulation of cellular process (55)	Single-multicellular organism process (25)
9	Cellular component organization (84)	Developmental process (53)	Response to stimulus (24)
10	Cellular component organization or biogenesis (83)	Anatomical structure development (53)	Developmental process (22)
Muscle contraction	63	29	19
Metabolic process	75	45	20
Response to oxidative stress	7	2	1
Oxidative phosphorylation	5	2	2

Ten most frequent biological processes and a selection of processes hypothesized to be affected by ELAM are shown. Number of residues significantly altered are shown in parentheses and for selected processes. One-way ANOVA *p* < 0.05

### Correlations across proteomics datasets

**Fig. 5:**
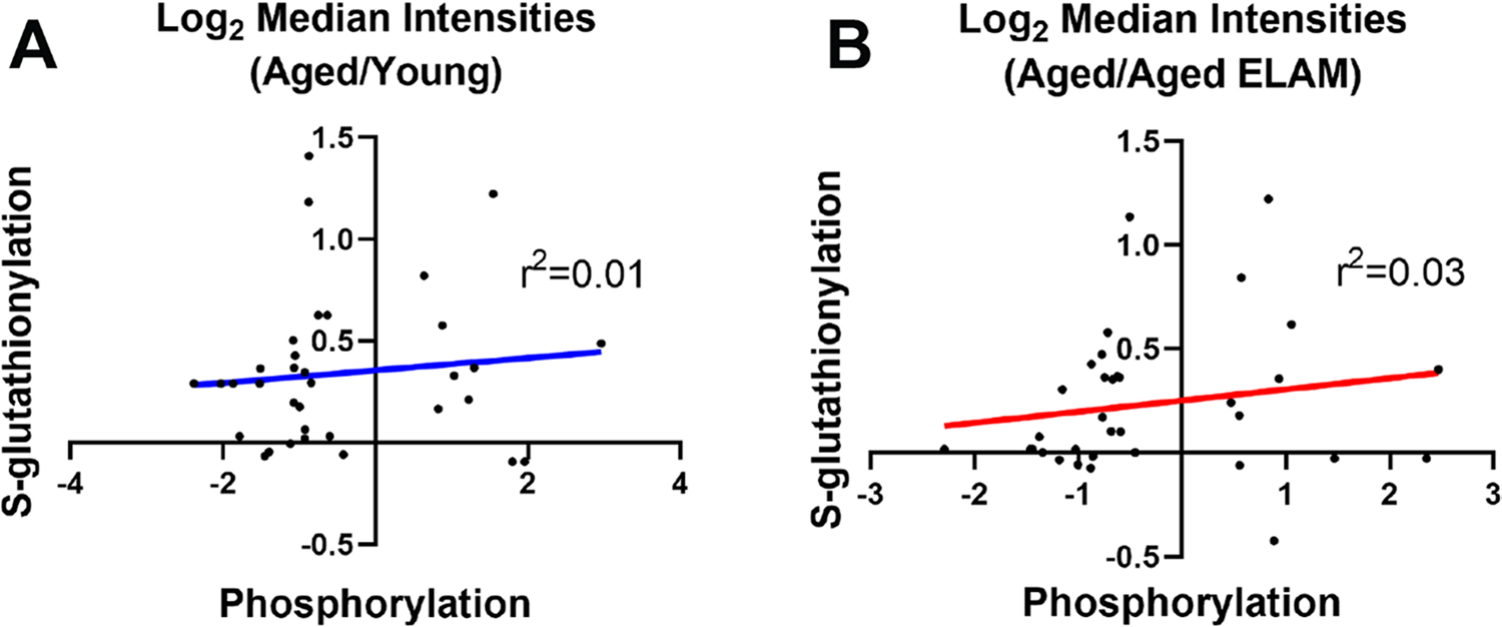
Comparison of phosphorylation site changes with changes in S-glutathionylation of nearest cysteine. **A** Aged/young comparison. **B** Aged/aged ELAM comparison. For proteins with multiple phosphorylation or S-glutathionylation sites, the closest cysteine residue to the relevant phosphorylation site was used

To test whether there was a link between redox changes and altered phosphorylation with age or ELAM, we analyzed the 38 residues in the phosphoproteome that were identified by ANOVA as changing with age and ELAM treatment (i.e., young/aged and aged/aged ELAM comparisons) and compared them to the nearest cysteine residue to identify possible interactions between S-glutathionylation and phosphorylation (Supplemental Table 1). There was no correlation between change in phosphorylation state of a given site and the change in S-glutathionylation of its nearest cysteine residue (Fig. 5).

Of the 38 sites in the phosphoproteome that were significantly altered in both aged/young and aged/aged ELAM comparisons, 12 proteins did not contain cysteine residues with significant S-glutathionylation changes. The 26 remaining phosphorylation residues did not show a clear pattern of nearby cysteine S-glutathionylation. While some phosphorylated sites were in close proximity to significantly altered S-glutathionylated cysteine residues, as little as four residues away in the case of L-lactate dehydrogenase A chain (LDHA), the majority of phosphorylated residues did not have an obvious nearby cysteine residue. The average distance between a significantly altered phosphorylated serine, tyrosine, or threonine and the nearest S-glutathionylated cysteine was approximately 359 amino acids (Supplemental Table 1). This does not rule out the possibility that a three-dimensional folded protein brings a cysteine residue into closer proximity to a phosphorylation site to act as a regulated post-translational modifier.

## Discussion

Treatment with ELAM has a unique effect on post-translational modifications as measured using proteomics. We have previously shown that ELAM strongly restores protein S-glutathionylation redox status in muscle of aged female mice. As protein S-glutathionylation can impact signaling via phosphorylation [24, 25], we chose to investigate if the profound effect of ELAM on S-glutathionylation proteomics extends to the phosphoproteome. We used a combination of abundance, S-glutathionylation, and phosphoproteomics to investigate possible links between the functional improvements caused by treatment with ELAM and proteomic profiles. We have previously published summary results on the abundance and S-glutathionylation proteomics results [13]. This report analyzes these datasets in the context of new phosphoproteomics data from the same muscle allowing direct comparisons between residues identified in separate datasets for the first time. Similar to cardiac muscle [21], these results show little effect of ELAM treatment on abundance proteomics, a modest effect on the phosphoproteome, and a very robust effect on protein S-glutathionylation. In contrast to the changes in S-glutathionylation [13], there are relatively few changes with either age or ELAM treatment to the phosphoproteome.

Interestingly, many of the 38 phosphoproteome sites that were significantly altered in both aged/young and aged/aged ELAM comparisons are within the myofilaments and contraction-related proteins. This is partly due to titin and myosin-4 containing multiple phosphorylated sites, but also includes alpha-actinin3, troponin I, desmin, myosin-binding protein C, and Serca1. Functional effects of phosphorylation on contractility have been known for decades [26]. Of the 38 phosphorylation sites identified here, some have previously been identified by other high-throughput phosphorylation analyses, but it is unknown if these sites alter function or are regulatory in nature. Only two phosphorylation sites identified here have previously been shown to have functional relevance. Increased phosphorylation of serine residue 377 on ATP-dependent 6-phosphofructokinase (PKFM) increases catalytic activity [27]. In addition, phosphorylation of serine 112 was identified as a regulatory site for Yes-associated Protein (YAP), a protein involved in mechanotransduction of skeletal muscle [28]. The functional impact of other phosphorylation changes identified here is outside the scope of this study. Given the number of myofilament- and contraction-related proteins that show changes in phosphorylation, as well as the improvement of S-glutathionylation, it is possible that redox status may play a role in the regulation of phosphorylation with age and that changes in these sites contribute to improved skeletal muscle function by treatment with ELAM [13]. Phosphorylation of titin has been hypothesized to effect mechanical strength in cardiomyocytes [29] and partial folding of titin domains is known to be altered by the presence of oxidized glutathione [30]. Thus, changes in the phosphoproteome and functional improvements may be at least partially due to restoration of redox status by ELAM [13].

A particularly striking finding within the phosphoproteome results is that in the 38 residues identified by ANOVA as significantly altered in both comparisons, 37 of those sites are rescued toward young phosphorylation levels by the ELAM treatment. This result is irrespective of whether age increases or decreases phosphorylation compared to young muscle. Only serine 1576 on XIRP2 shows decreased serine phosphorylation with age that is further decreased by treatment with ELAM. XIRP2 is an important effector of angiotensin II signaling in cardiac muscle [31] that binds to F-actin [32, 33], and is associated with heart failure. While the exact function in skeletal muscle is unclear, XIRP2 is normally localized to the costameres [34] and to nascent myofibrils following muscle injury [35], suggesting a possible role in repair and remodeling. Our data suggests that treatment with ELAM does not rescue, and in fact further decreases phosphorylation of XIRP2 serine 1576 (XIRP2-S1576) in aged muscle. Currently, the regulatory and/or functional role of phosphorylation of XIRP2-S1576 is unknown [36].

## Conclusions

This study showed that ELAM tends to reverse some of the age-related changes in phosphorylation status in skeletal muscle, although these changes are more subtle than reported for thiol redox PTMs. Despite profound effects on protein S-glutathionylation by ELAM, there are much fewer changes to protein abundance and phosphorylation. Changes of phosphorylation noted here, while much more subtle, include a large number of the altered sites present in metabolism- and contraction-related proteins further supporting functional improvement by ELAM treatment shown previously such as increased fatigue resistance and in vivo ATP production [12, 13]. Furthermore, it should be noted that phosphorylation status of the proteome here was examined in a basal rested state. Future studies investigating post-translational modifications or proteome changes should investigate alterations following muscle contraction, exercise, and other physiological stressors likely to induce rapid transient changes to phosphorylation or S-glutathionylation.

## Supplementary Information

Below is the link to the electronic supplementary material.

Supplementary file1 (DOCX 32.9 KB)
