# Supplementary material for: Elamipretide effects on the skeletal muscle phosphoproteome in aged female mice
Source: GeroScience. 2022 Nov 2;44(6):2913–24. doi: 10.1007/s11357-022-00679-0 (PMC9768078; doi:10.1007/s11357-022-00679-0)
Supplement: Supplementary file 1 — Supplementary file1 (DOCX 32.9 KB) [file 11357_2022_679_MOESM1_ESM.docx]

**Supplemental Materials**

| **Protein** | **Phosphorylation Site** | **Change with Age** | **Nearest Cysteine Residue** | **Change with Age** | **Distance by Amino Acid** |
| --- | --- | --- | --- | --- | --- |
| **ACTN3** | S601 | Up | 787 | Up | 186 |
| **ACTN3** | S610 | Up | 787 | Up | 177 |
| **AT2A1** | S473 | Down | 420 | Up | 53 |
| **AT2A1** | T532 | Down | 561 | Up | 29 |
| **BAF** | T2(0:1),T3 | Up | 67 | Up | 64 |
| **BAG3** | S138 | Up | N/A | N/A | N/A |
| **BAG3** | S390 | Up | N/A | N/A | N/A |
| **DESM** | S68 | Up | N/A | N/A | N/A |
| **EF1D** | S138 | Up | 217 | Up | 79 |
| **FLIP1** | S979 | Up | N/A | N/A | N/A |
| **KPYM** | T129 | Down | 31 | Up | 98 |
| **LDHA** | S80 | Down | 84 | Up | 4 |
| **MYG** | S121 | Up | 67 | Up | 54 |
| **MYH4** | Y389 | Down | 480 | Up | 91 |
| **MYH4** | S1514 | Down | 1443 | Up | 71 |
| **MYH4** | S1574 | Down | 1443 | Up | 131 |
| **MYH4** | S1600 | Down | 1443 | Up | 157 |
| **MYH4** | S1726 | Down | 1443 | Up | 283 |
| **MYPC2** | S482 | Down | 423 | Up | 59 |
| **PGM1** | S509 | Down | 251 | Up | 258 |
| **PFKAM** | S377 | Down | 334 | Up | 43 |
| **PURB** | S6,S8 | Up | N/A | N/A | N/A |
| **SMTL2** | S250 | Down | N/A | N/A | N/A |
| **SMTL2** | S339 | Down | N/A | N/A | N/A |
| **SYNPO** | S433 | Up | 927 | Up | 504 |
| **SYP2L** | S175,S177 | Up | N/A | N/A | N/A |
| **TITIN** | S9144 | Down | 9280 | Up | 136 |
| **TITIN** | T15495 | Down | 15426 | Up | 69 |
| **TITIN** | T20167 | Down | 18422 | Up | 1745 |
| **TITIN** | S22370(0:3) | Down | 24485 | Up | 2115 |
| **TITIN** | S29598 | Down | 29870 | Up | 272 |
| **TITIN** | S33875 | Down | 32630 | Up | 1245 |
| **TITIN** | T34360 | Down | 32630 | Up | 1730 |
| **TNNI2** | S90 | Down | 65 | Up | 25 |
| **VDAC1** | S240 | Down | 245 | Up | 5 |
| **XIRP2** | S1576 | Down | N/A | N/A | N/A |
| **YAP1** | S112 | Up | N/A | N/A | N/A |
| **ZCH18** | S530 | Up | N/A | N/A | N/A |

**Supplemental Table 1.** Nearest significantly altered cysteine residue to phosphorylation site.
